# Etidronate prevents, but does not reverse, ectopic mineralization in a mouse model of pseudoxanthoma elasticum (*Abcc6^−/−^*)

**DOI:** 10.18632/oncotarget.10738

**Published:** 2018-07-20

**Authors:** Qiaoli Li, Joshua Kingman, John P. Sundberg, Michael A. Levine, Jouni Uitto

**Affiliations:** ^1^ Department of Dermatology and Cutaneous Biology, The Sidney Kimmel Medical College at Thomas Jefferson University, Philadelphia, PA, USA; ^2^ The Jackson Laboratory, Bar Harbor, ME, USA; ^3^ Division of Endocrinology and Diabetes, Children's Hospital of Philadelphia and University of Pennsylvania Perelman School of Medicine, Philadelphia, PA, USA

**Keywords:** pseudoxanthoma elasticum, ectopic mineralization, etidronate treatment, bisphosphonates, mouse model

## Abstract

Pseudoxanthoma elasticum (PXE) and generalized arterial calcification of infancy (GACI) are heritable disorders manifesting with ectopic tissue mineralization. Most cases of PXE and some cases of GACI are caused by mutations in the *ABCC6* gene, resulting in reduced plasma pyrophosphate (PPi) levels. There is no effective treatment for these disorders. It has been suggested that administration of bisphosphonates, stable and non-hydrolyzable PPi analogs, could counteract ectopic mineralization in these disorders. In this study we tested the potential efficacy of etidronate, a first generation bisphosphonate, on ectopic mineralization in the muzzle skin of *Abcc6^−/−^* mice, a model of PXE. The *Abcc6^−/−^* mice received subcutaneous injections of etidronate, 0.283 and 3.40 mg/kg per injection (0.01× and 0.12×), twice a week, in both prevention and reversal studies. Ectopic mineralization in the dermal sheath of vibrissae in muzzle skin was determined by histopathologic analysis and by direct chemical assay for calcium content. Subcutaneous injection of etidronate prevented ectopic mineralization but did not reverse existing mineralization. The effect of etidronate was accompanied by alterations in the trabecular bone microarchitecture, determined by micro-computed tomography. The results suggest that etidronate may offer a potential treatment modality for PXE and GACI caused by *ABCC6* mutations. Etidronate therapy should be initiated in PXE patients as soon as the diagnosis is made, with careful monitoring of potential side effects.

## INTRODUCTION

Pseudoxanthoma elasticum (PXE), an autosomal recessive genodermatosis, is characterized by ectopic deposition of calcium hydroxyapatite in the skin, eyes, and the cardiovascular system, with considerable morbidity and occasional early mortality (For review see [1, 2]). The majority of cases with the classic form of PXE result from biallelic inactivating mutations in the *ABCC6* gene. *ABCC6* mutations can also cause generalized arterial calcification of infancy (GACI), another autosomal recessive disorder characterized by severe ectopic mineralization of the cardiovascular system that begins during fetal development [3, 4]. *ABCC6* encodes a transmembrane efflux transporter, ABCC6, expressed primarily in the liver, to a lesser extent in the kidneys, and at very low levels, if at all, in tissues subject to ectopic mineralization [5, 6]. These observations, together with results from animal studies, have suggested that PXE is a metabolic disorder deficient in circulating factor(s) required physiologically to prevent ectopic mineralization [7, 8]. While the precise physiologic function of ABCC6 is currently unclear, recent studies have indicated that ABCC6 is required for release of ATP from hepatocytes to the extracellular milieu where it is readily converted enzymatically to inorganic pyrophosphate (PPi) and AMP by ectonucleotide pyrophosphatase/phosphodiesterase 1 (ENPP1), an enzyme encoded by *ENPP1* [9, 10]. Since PPi is physiologically a powerful anti-mineralization factor and proper PPi/Pi ratio in circulation is required to prevent ectopic mineralization, loss-of-function mutations in *ABCC6* result in a reduced extracellular concentration of ATP and lowered PPi/Pi ratio, which then allow ectopic mineralization to ensue in patients with PXE.

There is currently no effective or specific treatment for PXE. The observations of reduced plasma PPi levels in PXE have led to the suggestion that administration of PPi would encounter ectopic mineralization, but unfortunately, PPi is unstable with relatively short half-life in circulation [11]. Recent studies have suggested, however, that administration of stable PPi analogs, bisphosphonates, could be used for treatment of ectopic mineralization disorders [12]. Bisphosphonates are structural analogs of PPi in which the oxygen linkage between phosphonate (PO3) groups is replaced by a carbon. Bisphosphonates also have two side groups (R1, R2), and different substitutions at these side chains alter their pharmacologic characteristics, specificity and relative potency [13, 14]. Bisphosphonates have two principal modes of action on mineral metabolism: (a) They can display physicochemical anti-mineralization properties that inhibit through their ability to limit the growth of mineral crystals, and (b) they exhibit biochemical properties that enable them to inhibit osteoclast activity. The latter property is the premise of their use in treatment of conditions characterized by bone loss, particularly osteoporosis [15]. Different bisphosphonates have various degrees of anti-mineralization and anti-osteoclastic activities. The first generation bisphosphonates, such as etidronate (ETD), a non-nitrogen containing bisphosphonate, displays considerable anti-mineralization activity, but is a relatively low-potency inhibitor of osteoclasts. By contrast, the third generation, nitrogen-containing bisphosphonates, as exemplified by alendronate (AST), are significantly more potent inhibitors of osteoclasts than the non-nitrogen containing ones, but the low doses required for this effect preclude administration of these drugs in quantities sufficient to exert significant inhibition of mineralization.

We recently initiated studies to explore the potential of bisphosphonates for treatment of ectopic mineralization disorders by administration of ETD or AST to genetically engineered mutant mice depicting extensive cutaneous and vascular mineral deposits. In one set of studies, these bisphosphonates were administered orally or by subcutaneous injections to *Enpp1^asj^* mice, a model for GACI, a severe disorder in which the patients often die within the first year of life from complications of vascular calcification [16]. The results suggested that these two bisphosphonates, particularly ETD, were capable of preventing ectopic mineralization in the dermal sheath of vibrissae and in the aorta. Furthermore, preliminary studies using a mouse model for PXE, the *Abcc6^tm1JfK^* mice (referred hereon as *Abcc6^−/−^* mice), demonstrated that feeding of ETD reduced mineralization at the early stages of the process [17]. However, relatively high doses of ETD were needed, probably due to the fact that absorption of ETD from the gastrointestinal track is poor, typically 1% or less [14]. Furthermore, these studies did not address the question of whether bisphosphonates are capable of reversing the ectopic mineralization by enhancing resorption of pre-existing calcium hydroxyapatite.

The *Abcc6^−/−^* mice have been shown to have markedly reduced PPi/Pi plasma ratio due to decreased PPi levels but normal Pi and calcium serum concentrations. [9, and Li et al., unpublished]. In this study, we have tested the efficacy of ETD in both preventing the ectopic mineralization process and in potentially reversing the existing calcification by subcutaneous injection of ETD into *Abcc6* knockout mice.

## RESULTS

### Experimental design

The *Abcc6^−/−^* mouse recapitulates features of PXE by developing late onset (5–6 weeks of age) and slowly progressing mineralization of the dermal sheath of vibrissae in the skin, Bruch's membrane in the eye, and in the arterial blood vessels of the cardiovascular system [18]. The first site of mineralization is the dermal sheath of vibrissae, a connective tissue capsule surrounding the sinus adjacent to the hair bulb of vibrissae. We have previously shown that the mineralization of this dermal sheath is progressive and reflects the mineralization of arterial blood vessels, thus allowing us to monitor the temporal degree of mineralization in this mouse model by histopathology or direct chemical assay of calcium in biopsies of muzzle skin or by non-invasive micro-computed tomography (μCT) [19, 20].

In this study, two sets of experimental design were utilized to test the efficacy of ETD. In the first set of experiments (Set 1), the Prevention Study, *Abcc6^−/−^* mice were treated with ETD by subcutaneous injections twice per week, initiated at weaning at 4 weeks of age, *i.e.*, before mineralization develops, and continued for another 8 weeks. This experiment allowed us to determine whether ETD can prevent ectopic mineral deposition in these mice. The second set of experiments (Set 2), the Reversal Study, allowed the mice to grow up to 12 weeks of age, *i.e.*, the age at which clearly detectable ectopic mineralization is present. The mice were then subjected to ETD administration by subcutaneous injections for another 12 weeks. In both sets of experiments, two doses of ETD were used, 0.283 and 3.40 mg per kg of body weight per injection; these concentrations correspond to 1 percent (0.01×) and 12 percent (0.12×) of the typical oral dose used for treatment of osteoporosis in adult human patients (8 mg/kg/day assuming 50 kg body weight; http://www.rxlist.com/didronel-drug.htm). For proper amount of ETD to be injected, the mice were weighed every week, and the dose was adjusted accordingly per g body weight. In both experiments, mice were treated in parallel with saline injections. Each group consisted of 7–10 mice, with approximately equal distribution between males and females (Table 1).

**Table 1:** Experimental groups of *Abcc6^−/−^* mice by genotype and treatment^*^

Group, injected with	Genotype	No. of mice examined (M+F)	Treatment started > treatment ended (weeks)
Prevention Study (Set 1):			
saline	*Abcc6^−/−^*	7 (3+4)	4 > 12
0.01× ETD	*Abcc6^−/−^*	10 (5+5)	4 > 12
0.12× ETD	*Abcc6^−/−^*	10 (6+4)	4 > 12
NT	*Abcc6^−/−^*	10 (5+5)	4 > 12
Reversal Study (Set 2):			
saline	*Abcc6^−/−^*	10 (5+5)	12 > 24
0.01× ETD	*Abcc6^−/−^*	10 (5+5)	12 > 24
0.12× ETD	*Abcc6^−/−^*	10 (5+5)	12 > 24
NT	*Abcc6^−/−^*	10 (5+5)	12 > 24
NT	*Abcc6*^+/+^	10 (5+5)	12 > 24

^*^ All the mice were placed on control diet at 4 weeks of age post-weaning. In Prevention Study, the mice at 4 weeks of age were injected subcutaneously with saline, 0.01× ETD or 0.12× ETD, for an additional 8 weeks. Mineralization was determined at 12 weeks of age. In Reversal Study, the *Abcc6^−/−^* mice were allowed to develop mineralization up to 12 weeks of age and they were then injected and followed for an additional 12 weeks. At this point, at 24 weeks of age, the mice were euthanized for analysis. ETD, etidronate; M, male; F, female; NT, not treated.

At the end of each experiment, the mice were sacrificed, and the degree of mineralization in the dermal sheath of vibrissae was determined semi-quantitatively by staining tissue sections with Alizarin red or by direct chemical assay of calcium content in the biopsies of muzzle skin containing the dermal sheath of vibrissae. Since ETD is known to affect the morphology of long bones, the femurs of these mice were examined separately in male and female mice by μCT.

### Etidronate prevents ectopic mineralization in the skin

**Figure 1:**
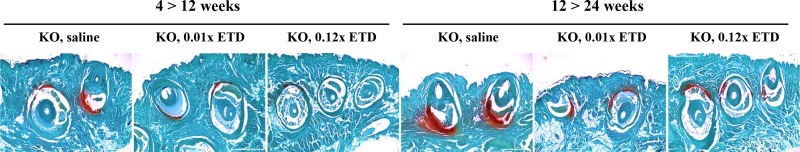
Histopathology with Alizarin red stain demonstrates that etidronate treatment prevents, but does not reverse, ectopic soft tissue mineralization in *Abcc6^−/−^* mice The *Abcc6^−/−^* mice develop ectopic mineralization of the dermal sheath of vibrissae prior to 12 weeks of age, and this process progresses to 24 weeks of age. Subcutaneous injection of etidronate at 0.01× and 0.12× doses during 4–12 weeks markedly reduced the degree of mineralization in the dermal sheath of vibrissae, when compared to age-matched *Abcc6^−/−^* mice receiving saline injections (left 3 panels). Etidronate treatment from 12 to 24 weeks of age prevented further mineralization but did not reverse mineralization that had developed prior to 12 weeks of age (right 3 panels). ETD, etidronate; KO, knockout. Scale bar = 400 μm.

**Figure 2:**
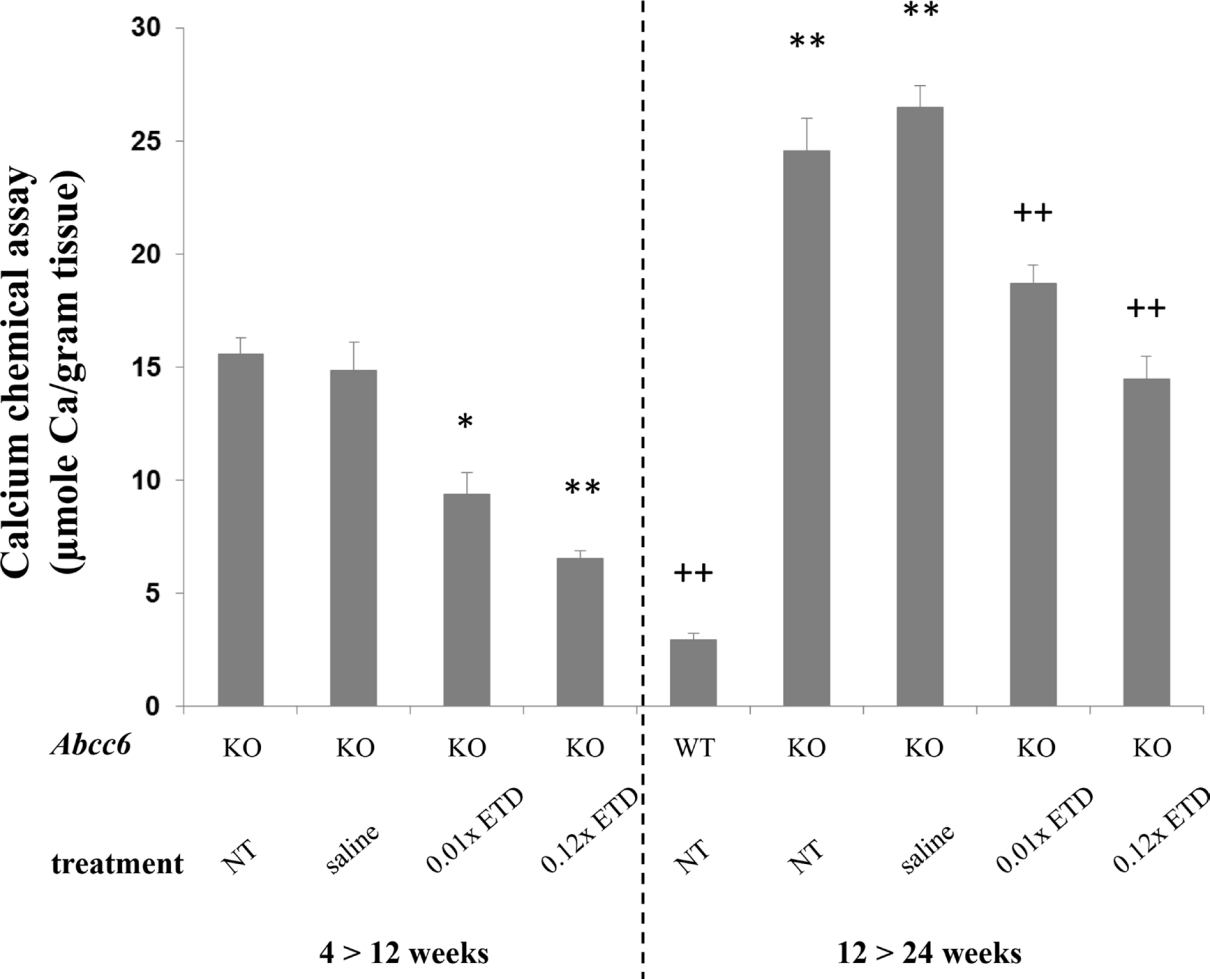
Etidronate treatment prevents vibrissae mineralization as determined by the direct chemical assay of calcium Note the significantly elevated calcium content in the muzzle skin of *Abcc6^−/−^* mice, either NT or injected with saline, as compared with the WT mice (right panel). Injections of *Abcc6^−/−^* mice subcutaneously with 0.01× and 0.12× ETD significantly reduced the calcium content of the muzzle skin as compared with age-matched mice injected with saline. Etidronate treated *Abcc6^−/−^* mice at 24 weeks of age demonstrated mineralization similar to *Abcc6^−/−^* mice at 12 weeks of age, indicating that ETD prevents further mineralization but does not reverse mineralization that had developed before initiation of treatment. ^*^p < 0.05, ^**^p < 0.01, compared to KO mice at 12 weeks of age; ^++^*p* < 0.01, compared to KO mice at 24 weeks of age. Mean ± SE; *n* = 7–10 mice per group. ETD, etidronate; KO, knockout; WT, wild type; NT, not treated.

In the first set of experiments, ETD or saline injections were initiated at 4 weeks of age, a time point at which no ectopic mineralization is as yet present. At 12 weeks of age, *Abcc6^−/−^* mice that had been injected with saline revealed extensive ectopic mineralization in the dermal sheath of vibrissae (Figure 1). Injection of either 0.01× ETD or 0.12× ETD significantly reduced the degree of mineralization as determined semi-quantitatively by histopathologic examination (Figure 1) or by direct quantitative assay of calcium content of the muzzle skin (Figure 2). Wild type mice on the corresponding strain background (C57BL/6J) did not develop ectopic mineralization in the vibrissae (not shown).

### Etidronate does not reverse the existing ectopic mineralization in *Abcc6^−/−^* mice

Examination of the dermal sheath of vibrissae of the *Abcc6^−/−^* mice that were untreated controls or were injected with saline at 24 weeks of life demonstrated progressive mineralization as compared to mice at 12 weeks of age (Figures 1 and 2). Injection of ETD, 0.01× or 0.12× between 12 and 24 weeks of age, resulted in lower level of mineralization than in mice injected with saline. However, the degree of mineralization, as quantitated by chemical assay of calcium content, in ETD treated mice at 24 weeks of age was of the same order of magnitude as in those injected with saline at 12 weeks of age. These results indicate that ETD administration did not resolve the mineral deposits that had accumulated in the mice before the treatment was started at 12 weeks of age as shown in Set 1. Rather, further mineralization in Set 2 was arrested at the level that existed at 12 weeks of age but did not lower the value below the baseline (Figure 2).

### Etidronate modifies the bone microarchitecture

**Figure 3:**
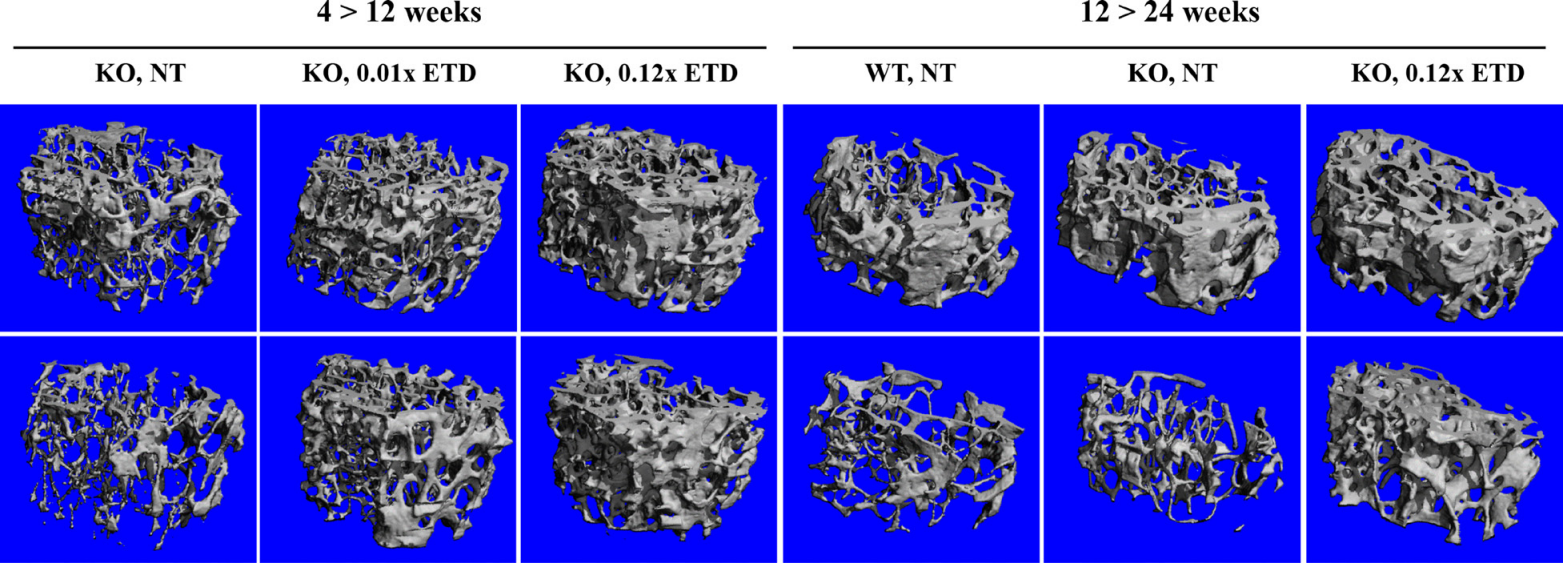
Etidronate treatment of *Abcc6^−/−^* mice results in altered femur microarchitecture Note the distinct difference between male (upper panel) and female (lower panel) mice, assessed by μCT scan. Treatment with etidronate caused changes in femur microarchitecture, as quantitatively detailed in Table 2. Three males and 3 females were examined in each group with similar findings. ETD, etidronate; KO, knockout; WT, wild type; NT, not treated.

Because ETD possesses both anti-mineralization and anti-osteoclastic activities, we further evaluated the morphometric microarchitecture of the trabecular bone in the ETD treated mice by μCT analysis of their femurs. Marked differences in the bone density and trabecular microarchitecture were noted between the male and female mice, consistent with previous reports [16, 17, 21, 22]. Consequently, sex-matched comparisons of the μCT results were performed between *Abcc6^−/−^* mice and the corresponding wild type mice on the same strain background as well as with mice treated with 0.01× or 0.12× ETD in both sets of experiments. No significant differences in the bone microarchitecture between the *Abcc6^−/−^* mice and the corresponding, sex-matched wild type mice were noted (Table 2). However, treatment of the mice with ETD resulted in marked increase in the bone mass (Figure 3). These changes of bone microarchitecture were quantified using manufacturer-provided software (Table 2). In treated mice, the distal femoral bone volume fraction (BV/TV, %) was increased, corresponding to the increased trabecular bone mineral density (BMD). ETD treatment also caused increased trabecular number (Tb.N), decreased trabecular separation (Tb.Sp), decreased structure model index (SMI) and connectivity density (Conn.D). These results attest to the potency of ETD in anti-osteoclastic activities directed at bone.

**Table 2:** Trabecular bone phenotypes by microCT of the right distal femur of the mice^*^

Group, injected with	Sex	BMD (mg/cm^3^)	BV/TV (%)	Tb.Th (μm)	Tb.N (1/mm)	Tb.Sp (μm)	SMI	Conn.D (TV/mm^3^)
Prevention Study (Set 1):								
KO, saline	M	988.1 ± 8.2	8.9 ± 1.1	33.6 ± 1.9	4.7 ± 0.1	207.5 ± 7.2	2.3 ± 0.2	222.6 ± 34.7
KO, saline	F	963.4 ± 5.2	5.2 ± 0.4	29.7 ± 0.2	4.0 ± 0.1	246.4 ± 3.0	2.7 ± 0.1	124.0 ± 30.4
KO, 0.01× ETD	M	1064.5 ± 30.9^+^	9.8 ± 1.3	34.1 ± 0.6	5.3 ± 0.1^+^	212.1 ± 18.0	2.1 ± 0.1	250.7 ± 46.5
KO, 0.01× ETD	F	999.5 ± 7.1^+^	10.2 ± 1.2^+^	33.3 ± 1.5	4.9 ± 0.3^+^	197.3 ± 12.3^+^	2.0 ± 0.2^+^	282.5 ± 37.9^+^
KO, 0.12× ETD	M	1023.4 ± 1.8^+^	18.0 ± 1.5^++^	36.5 ± 0.7	6.0 ± 0.2^++^	156.6 ± 6.9^++^	1.1 ± 0.2^+^	402.0 ± 28.0^+^
KO, 0.12× ETD	F	1146.6 ± 67.0^+^	15.1 ± 1.6^++^	34.4 ± 1.2^+^	5.5 ± 0.5^+^	177.5 ± 16.2^+^	1.4 ± 0.2^++^	400.6 ± 73.9^+^
Reversal Study (Set 2):								
KO, saline	M	993.1 ± 7.5	8.7 ± 1.2	35.2 ± 2.5	4.3 ± 0.1	224.9 ± 3.8	2.1 ± 0.2	169.5 ± 13.8
KO, saline	F	981.0 ± 2.6	3.4 ± 0.3	33.7 ± 1.1	2.9 ± 0.1	345.2 ± 10.0	2.9 ± 0.1	53.6 ± 8.5
KO, NT	M	994.4 ± 9.2	12.8 ± 3.4	41.6 ± 5.0	4.4 ± 0.2	219.0 ± 13.0	1.7 ± 0.3	166.1 ± 8.4
KO, NT	F	988.0 ± 11.2	4.2 ± 0.5	32.7 ± 1.2	3.2 ± 0.1	316.7 ± 12.5	2.5 ± 0.1	81.1 ± 8.4
KO, 0.12× ETD	M	1033.2 ± 10.5^+^	20.4 ± 2.7	44.6 ± 1.1	5.2 ± 0.3	184.1 ± 13.3	0.8 ± 0.2	220.4 ± 16.3^+^
KO, 0.12× ETD	F	982.1 ± 11.3	12.5 ± 1.2^++^	38.9 ± 1.4^+^	3.9 ± 0.1^++^	250.3 ± 6.1^++^	1.3 ± 0.1^++^	167.5 ± 11.5^++^
WT, NT	M	1003.8 ± 10.2	13.6 ± 0.1	48.2 ± 1.0	4.2 ± 0.2	226.2 ± 8.8	1.7 ± 0.2	140.2 ± 11.8
WT, NT	F	1003.3 ± 1.6	3.8 ± 0.8	34.8 ± 0.4	3.0 ± 0.2	335.0 ± 19.8	2.8 ± 0.1	59.5 ± 17.9

^*^For a description of different groups, see Table 1. Values are expressed as mean ± SE (three mice of each sex per group). Statistical significance in comparison to age-matched untreated KO mice of the same sex is indicated: ^+^*p* < 0.05, ^++^*p* < 0.01. BMD, bone mineral density (mg/cm^3^); BV/TV, relative bone volume (%); Tb.Th, trabecular thickness (μm); Tb.N, trabecular number (1/mm); Tb.Sp, trabecular separation (marrow thickness, μm); SMI, structure model index; Conn.D, connectivity density (TV/mm^3^). ETD, etidronate; M, male; F, female; NT, not treated

## DISCUSSION

**Figure 4:**
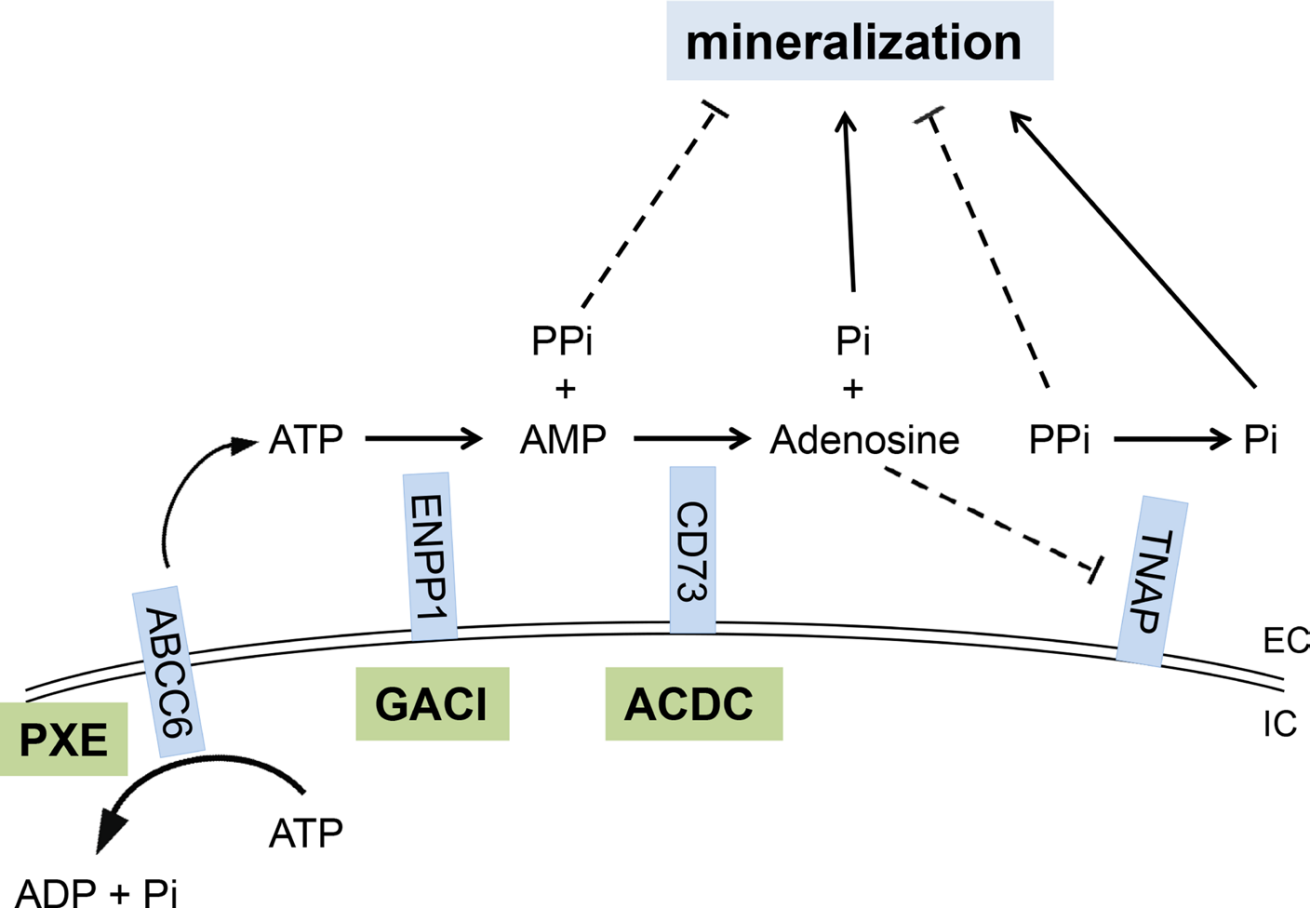
The PPi and Pi generating pathway points to the critical role of components of the pro-mineralization/anti-mineralization network Mutations in the *ABCC6*, *ENPP1*, and *NT5E* genes cause pseudoxanthoma elasticum (PXE), generalized arterial calcification of infancy (GACI), and arterial calcification due to CD73 deficiency (ACDC), respectively. ABCC6, a putative transmembrane transporter, mediates ATP release from hepatocytes to extracellular space where ATP is converted to PPi and AMP by ENPP1, a membrane-bound pyrophosphatase/phosphodiesterase. CD73 converts AMP to Pi and adenosine, the latter one being an inhibitor of tissue nonspecific alkaline phosphatase (TNAP), an extracellular, yet membrane-bound protein, that hydrolyze PPi to Pi. PPi is an anti-mineralization factor, and Pi is a pro-mineralization factor. Deficiencies in ABCC6, ENPP1 and CD73 proteins lead to reduced plasma PPi levels and PPi/Pi ratio, thereby promoting mineralization in peripheral tissues. EC, extracellular; IC, intracellular.

PXE, a prototype of heritable ectopic mineralization disorders, manifests with progressive deposition of calcium hydroxyapatite crystals in soft connective tissues, particularly in the skin and the cardiovascular system. The disease is of late-onset, and the average age at diagnosis is in early teens, although in some patients the diagnosis is not reached until in their late thirties or forties [1, 2]. GACI is clinically overlapping, yet more severe, ectopic mineralization disorder, the majority of patients dying from cardiovascular complications during their first year of life [23, 24]. Another, recently described ectopic mineralization disorder is arterial calcification due to CD73 deficiency (ACDC) which manifests with late-onset of mineralization affecting primarily the arterial blood vessels in the lower extremities and the periarticular ligaments and tendons [25]. The genetic basis of these three conditions resides within a pathway involved in generation of PPi and Pi, the mutations in classic PXE being in *ABCC6*, in GACI in *ENPP1*, and in ACDC in *NT5E* (Figure 4) [1, 26]. Thus, the unifying pathogenetic feature of these conditions is reduced plasma PPi/Pi ratio which allows ectopic mineralization in peripheral tissues to ensue [1, 9, 23, 25].

A number of spontaneous and genetically engineered mutant mice have been characterized with ectopic mineralization of soft connective tissues in the skin and vascular connective tissues, and many of them recapitulate features of PXE, GACI and ACDC, respectively [26, 27]. In a particular, the *Abcc6^−/−^* mouse serves as an appropriate model for PXE, characterized by late-onset mineralization of skin, eyes, and the arterial blood vessels [18, 28]. Examination of these mice by energy dispersive X-ray and Fourier transform infrared imaging spectroscopy (FT-IRIS) indicated that the principal components of the mineral deposits in tissues are calcium and phosphate, and the Ca/P ratio increases in samples with progressive mineralization reaching a value of close to 2:1, comparable to that in endochondral bone [29]. Progressively increasing mineralization is also reflected by increased mineral-to-matrix ratio, and the determination of the mineralization process by FT-IRIS suggested progressive maturation of the mineral deposits from amorphous calcium phosphate to hydroxyapatite [29]. In this study, we utilized *Abcc6^−/−^* mice as a model for PXE to test the potential efficacy of ETD, a stable PPi analog. Bisphosphonates, such as ETD, can prevent ectopic mineralization by incorporation into calcium hydroxyapatite mineral crystals preventing their growth [30].

There is no specific or effective treatment for PXE, and previous clinical trials attempting to counteract the mineralization process, such as increasing the magnesium content of the diet or feeding phosphate binders, have highlighted the challenges of the trials in these patients with slowly progressing and unpredictable clinical progression [31, 32]. Such clinical trials are further complicated by considerable phenotypic, both intra- and inter-familial, variability which may be compounded by modifying genetic factors and life style variables, such as diet and exercise [33]. In this study, we have specifically tested the potential of ETD as a treatment of PXE under carefully controlled laboratory conditions on a genetically defined mouse strain background. Our results demonstrated that ETD, at the dosages used was able to prevent ectopic mineralization in mice when started before the onset of the mineralization process, as demonstrated by our Prevention Study. However, once the mineral deposits had accumulated in soft connective tissue, ETD did not reverse the extent of mineralization but arrested further deposition of mineral at the point when the treatment was initiated. These observations would imply that if ETD is to be used to treat patients with PXE, it is ideally started as soon as the diagnosis has been established. In this regard, a pre-symptomatic mutation analysis can establish the diagnosis of PXE in asymptomatic patients in families with previous history of PXE [34]. Therefore, one would recommend that all siblings, particularly younger ones, of patients diagnosed with PXE should be examined for *ABCC6* mutations to establish their genotypic status.

Because ETD has previously shown to be effective for treatment of disorders characterized by loss of bone mass, such as osteoporosis and osteogenesis imperfecta but also bone destruction by cancer metastases [15, 35], we determined the morphometric characteristics of bones in PXE mice with and without treatment with ETD. The results demonstrated that untreated PXE mice, at least up to 24 weeks of age, did not show differences in bone morphology as compared with wild type control mice of the same genetic strain background. Whether such findings are applicable to PXE patients as well remains to be determined by bone scanning analyses in patients. Treatment of *Abcc6^−/−^* mice with ETD resulted in significant increases in bone mass, as determined by μCT. These results indicated that ETD in dosages used in our study was effective in modifying the bone density, and they also suggested that ETD could potentially have dual beneficial effects in patients with PXE both by preventing ectopic mineralization and increasing bone mass as part of prevention of osteoporosis.

Recently (May, 2015), a clinical trial for treatment of ectopic mineralization in PXE was registered in the Netherland's Trial Registry (NTR5180; http://www.trialregister.nl/trialreg/admin/rctview.asp?TC=5180). This randomized, double-blinded and placebo controlled study proposes to test the efficacy of ETD (20 mg/kg per day) cycling for 2 weeks on and 10 weeks off for 12 months compared with placebo. The primary end point is the degree of mineralization in the leg arteries after 12 months of treatment as measured by ^18^F-NaF uptake. Secondary objectives include the effects of ETD therapy on calcium scores of the peripheral arteries, attenuation of ongoing calcification in other arteries than leg arteries, and ophthalmological and dermatological changes. Based on our preclinical mouse studies reported here, one would predict that ETD in this clinical study will attenuate the progression of mineralization but does not reverse the existing level of mineralization with accompanied clinical manifestations. This prediction is based on the assumption that the efficacy, pharmacokinetics, and metabolism of ETD are similar in mice and humans. Indeed, it will be interesting to see whether ETD treatment in this clinical trial will show beneficial effects to patients with PXE. These clinical trials should also consider the established risks associated with bisphosphonate treatment, such as bone pain or tenderness, osteonecrosis and bone fractures [36]. Prolonged continued treatment has also been reported to cause nephrotic syndrome. In addition to PXE, a study on a case series of patients with GACI suggested that bisphosphonate treatment of newborns with this disease was associated with the survival beyond infancy, although the precise mechanism of this effect was not established [12]. Finally, a clinical trial proposes to treat patients with ACDC with ETD (NCT01585402; https://clinicaltrials.gov/show/NCT01585402) and is currently recruiting participants. Thus, all these conditions of ectopic mineralization presenting with reduced PPi/Pi ratio may be candidates for bisphosphonate treatments, particularly ETD, to counteract the clinical manifestations. Finally, it should be noted that other approaches to increase the PPi/Pi ratio in patients with ectopic mineralization disorders should be considered, including increased release of ATP to the extracellular milieau, enhancement of ENPP1 activity [37], and inhibition of tissue non-specific alkaline phosphatase (TNAP) [38] (see Figure 4), towards development of molecularly designed pharmacologic agents with anti-mineralization potential.

## MATERIALS AND METHODS

### Mice

The *Abcc6^tm1JfK^* mouse was developed by targeted ablation of the *Abcc6* gene (this mouse is referred to as *Abcc6^−/−^*) [18]. The *Abcc6* null allele was backcrossed for ten generations into the C57BL/6J strain to create a congenic strain (B6.Cg). *Abcc6* wild type (*Abcc6*^+/+^) and knockout (*Abcc6^−/−^*) mice were generated from heterozygous breeding. Mice were fed a standard rodent diet (Lab Diet 5010; PMI Nutrition, Brentwood, MO, USA) with free access to water. Mice were maintained in the Animal Facility of Thomas Jefferson University in a temperature- and humidity-controlled environment. All protocols were approved by the Institutional Animal Care and Use Committee of Thomas Jefferson University. Proper handling and care were practiced according to the animal welfare policies of the United States Public Health Service.

### Experimental design and treatment

WT and *Abcc6^−/−^* mice were placed on the standard rodent diet throughout the experiment. Mice were injected with saline or etidronate (ETD) subcutaneously, twice a week. Mice were divided into four groups: (i) mice were not treated; mice were injected with (ii) sterile saline, (iii) 0.01× ETD, or (iv) 0.12× ETD. The 1× dose was calculated to be equivalent to that used for the treatment of humans for osteoporosis (8 mg/kg/day orally). The dose of 0.01× ETD was based on the assumption of 1% of intestinal absorption of ETD, whereas 100% absorption by subcutaneous injection [13, 14, 39]. Mice were assigned into the Prevention Study (Set 1) and the Reversal Study (Set 2) groups with appropriate controls. The groups of mice in the two sets, characterized by genotype and treatment, are described in Table 1. In Set 1, *Abcc6^−/−^* mice were injected with ETD beginning at 4 weeks of age for a total of 8 weeks, 7–10 mice per group. All mice were euthanized at 12 weeks of age for tissue analysis. In Set 2, *Abcc6^−/−^* mice were injected with ETD beginning at 12 weeks of age for a total of 12 weeks, 10 mice per group, and were euthanized at 24 weeks of age for tissue analysis.

### Histopathological analysis

Biopsies from muzzle skin containing vibrissae were fixed in 10% phosphate-buffered formalin and embedded in paraffin. Paraffin sections (6 μm thick) were stained with Hematoxylin and Eosin and Alizarin red using standard methods. Slides were examined under light microscopy for mineralization.

### Chemical quantitation of calcium in the muzzle skin

To quantify calcium deposition in vibrissae, muzzle skin biopsies were decalcified with 0.15 mol/L HCl for 2 days at room temperature. Solubilized calcium was then determined by a colorimetric assay using the o'-cresolphthalein complexone method [calcium (CPC) LiquiColor; Stanbio Laboratory, Boerne, TX]. The amount of calcium was normalized to tissue weight.

### Micro-computed tomography

Microarchitecture of the distal trabecular bone of the right femur was determined in a 1.25 mm-thick region located proximal to the distal growth plate of femur by scanning at a 10.5 μm resolution using the micro-computed tomography (μCT) system (vivaCT40; Scanco Medical AG, Bassersdorf, Switzerland). The microstructural parameters were obtained through 3-dimensional reconstruction using a Gaussian filter and a global threshold of 2272 Hounsfield units in the manufacturer-provided software.

### Statistical analysis

We performed comparisons between different groups of mice using the 2-sided Kruskal–Wallis nonparametric test. Statistical significance was reached with *p* < 0.05. All statistical computations were completed using SPSS version 15.0 software (SPSS Inc., Chicago, IL).

## Abbreviations

PXE: pseudoxanthoma elasticum
GACI: generalized arterial calcification of infancy
ETD: etidronate
AST: alendronate
KO: knockout
WT: wild type
PPi: inorganic pyrophosphate
Pi: inorganic phosphate
NT: not treated
